# Supplementary figures and images for: Predefined and data-driven CT radiomics predict recurrence-free and overall survival in patients with pulmonary metastases treated with stereotactic body radiotherapy
Source: PLoS One. 2024 Dec 31;19(12):e0311910. doi: 10.1371/journal.pone.0311910 (PMC11687728; doi:10.1371/journal.pone.0311910)

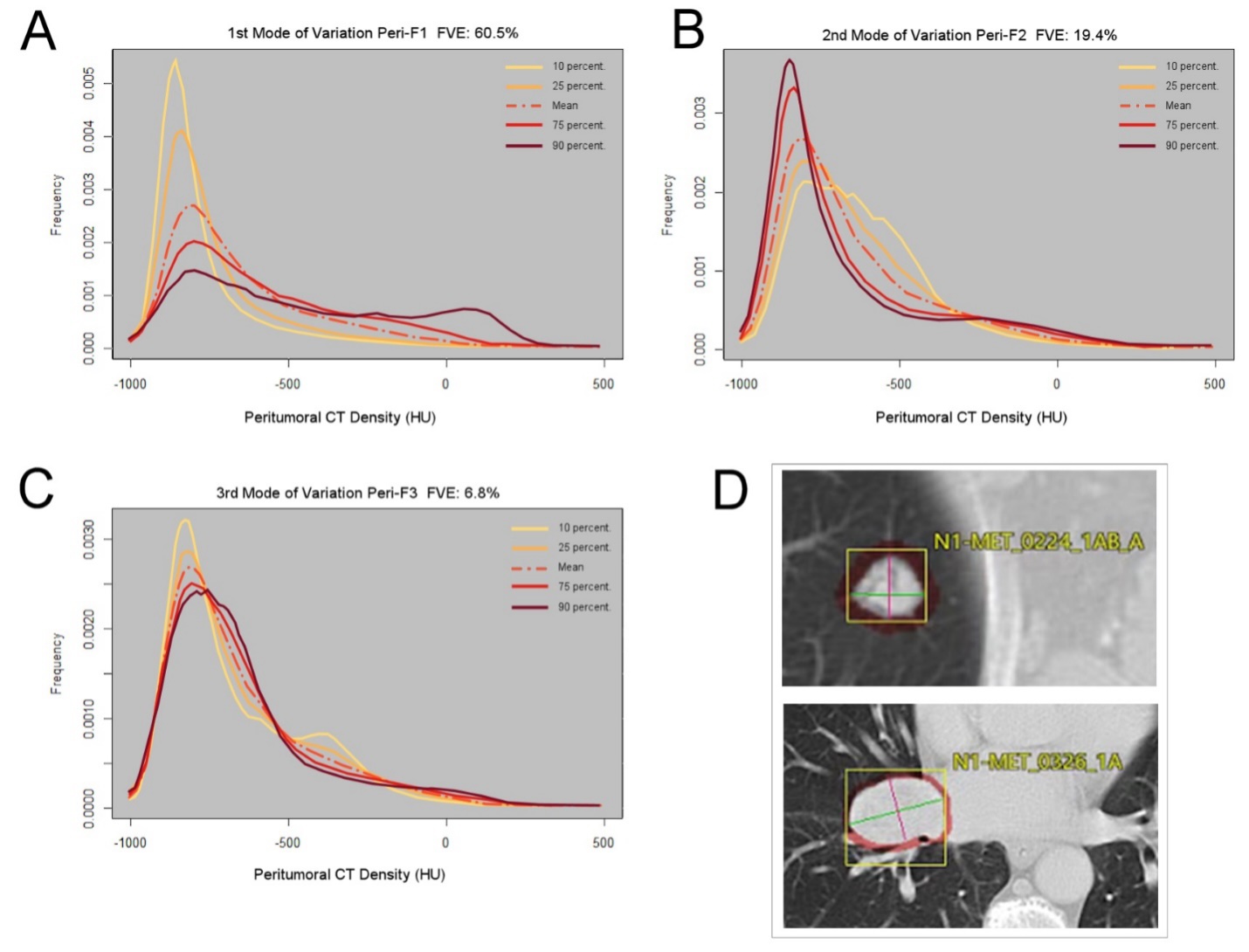

Supplement: S1 Fig — A. First main mode variation Peri-F1 of the peri-tumoral CT density histogram from low density homogeneous distribution (yellow) to heterogeneous bimodal distribution (brown). B. Second mode of variation Peri-F2. C. Third mode of variation Peri-F3. D. Example of peri-tumoral region with low Peri-F1 (top) and high Peri-F1 (bottom) (Vitrea software v.7.6, Canon Medical systems, Otawara, Japan). (JPG) [file pone.0311910.s001.jpg]

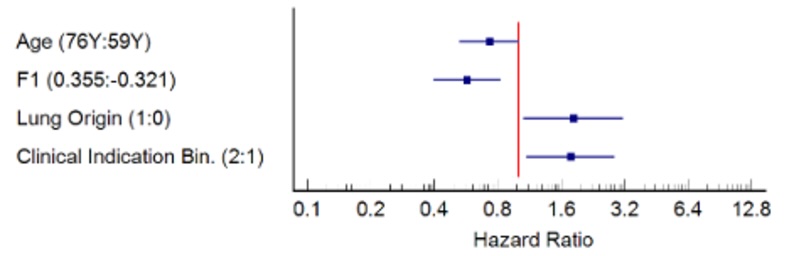

Supplement: S2 Fig — Hazard ratios are presented with their 95% confidence intervals. (JPG) [file pone.0311910.s002.jpg]

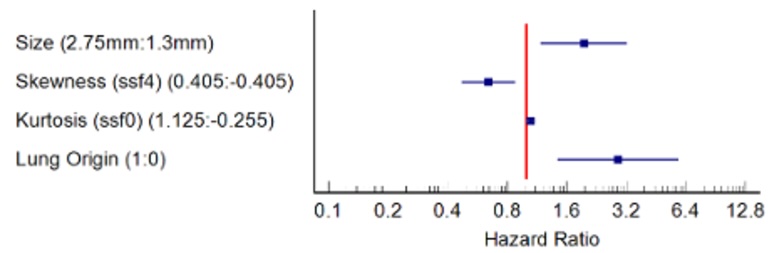

Supplement: S3 Fig — Hazard ratios are presented with their 95% confidence intervals. (JPG) [file pone.0311910.s003.jpg]

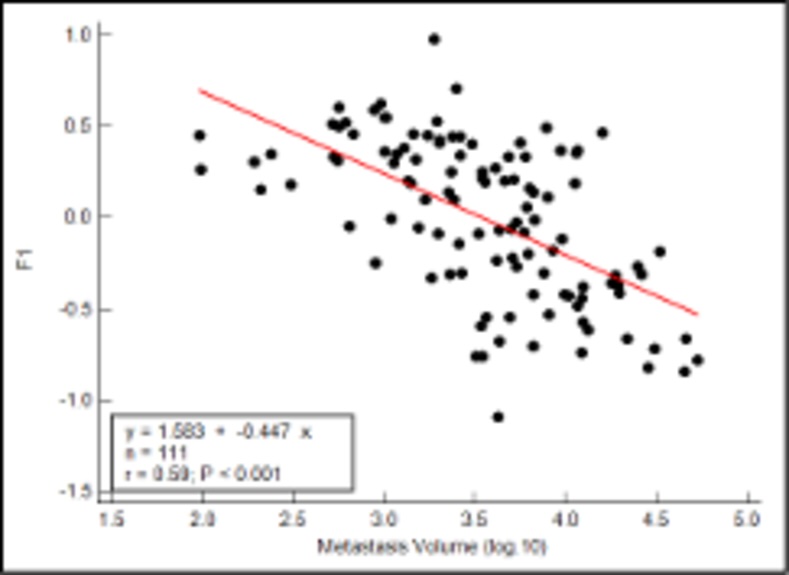

Supplement: S4 Fig — The CT density variable F1 appears fairly correlated with the metastasis volume (log). Linear correlation r = 0.59. (JPG) [file pone.0311910.s004.jpg]

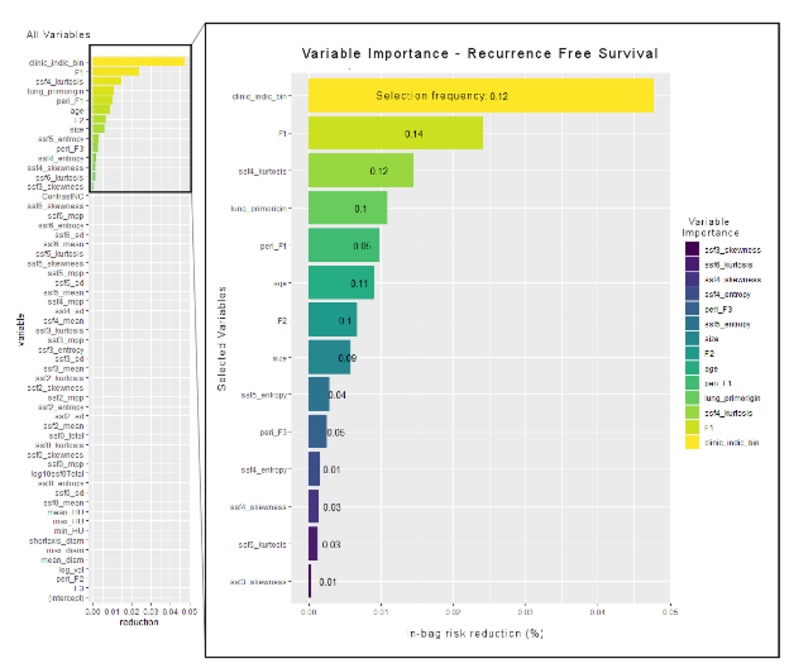

Supplement: S5 Fig — Left: Original variable list (including clinical variables). Right: Selected variables for Recurrence Free Survival (RFS) multivariate Cox model building after 100 boosting iterations ranked by decreasing importance (% in-bag reduction risk). (JPG) [file pone.0311910.s005.jpg]

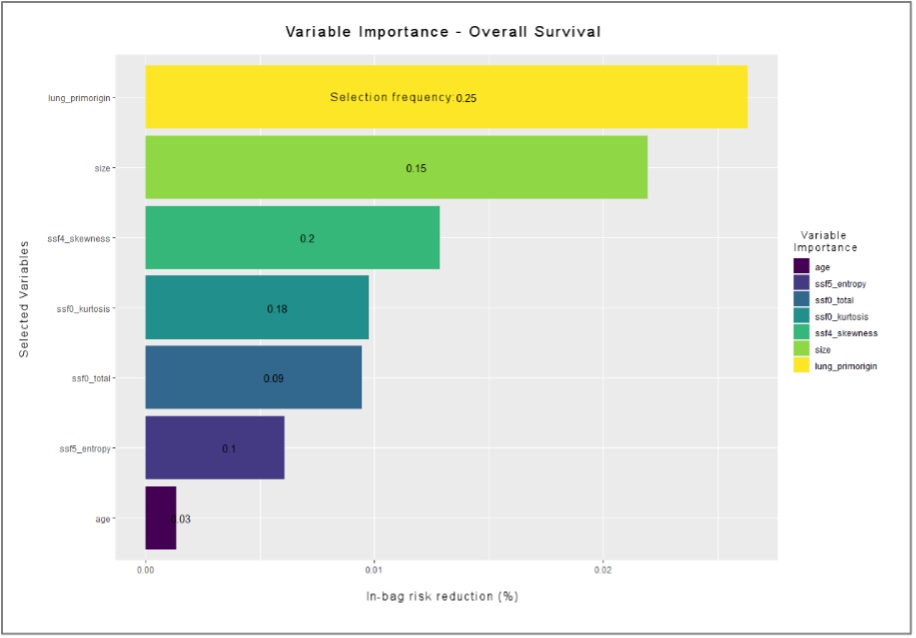

Supplement: S6 Fig — Left: Original variable list (including clinical variables). Right: Selected variables for Recurrence Free Survival (RFS) multivariate Cox model building after 100 boosting iterations ranked by decreasing importance (% in-bag reduction risk). (JPG) [file pone.0311910.s006.jpg]
