# Supplementary material for: Predefined and data-driven CT radiomics predict recurrence-free and overall survival in patients with pulmonary metastases treated with stereotactic body radiotherapy
Source: PLoS One. 2024 Dec 31;19(12):e0311910. doi: 10.1371/journal.pone.0311910 (PMC11687728; doi:10.1371/journal.pone.0311910)
Supplement: S1 File — (DOCX) [file pone.0311910.s007.docx]

**Supplementary Material**

**Predefined and data-driven CT radiomics predict recurrence-free and overall survival in patients with pulmonary metastases treated with stereotactic body radiotherapy**

Pascal Salazar PhD^1^, Patrick Cheung MD FRCPC^2^, Balaji Ganeshan PhD^3^, Anastasia Oikonomou MD PhD FRCPC^4^

^1^Canon Medical Systems, Minnetonka, MN 55343, USA

^2^Department of Radiation Oncology, Sunnybrook Health Sciences Centre, University of Toronto, 2074 Bayview Avenue, Toronto, Ontario, Canada, M4N 3M5

^3^Institute of Nuclear Medicine, University College London, 235 Euston Road, London NW1 2BU, UK

^4^Department of Medical Imaging, Sunnybrook health Sciences Centre, University of Toronto, 2074 Bayview Avenue, Toronto, Ontario, Canada, M4N 3M5

Corresponding Author

Anastasia Oikonomou MD PhD FRCPC

Department of Medical Imaging, Sunnybrook health Sciences Centre, University of Toronto

2075 Bayview Avenue, Toronto, Ontario, Canada, M4N 3M5

email: anastasia.oikonomou@sunnybrook.ca

**SBRT technique**

The SBRT technique at our institution has been previously described^1^. Patients were immobilized using one of 2 techniques: the Elekta BlueBAG vacuum cushion (Elekta AB, Stockholm, Sweden) with an abdominal compression plate, or the full Elekta BodyFIX system. A four dimensional (4D)-CT was acquired with phase-binning reconstruction software. The gross tumor volume (GTV) was delineated by the radiation oncologist on the 0% (peak inspiratory), 50% (peak expiratory), and maximum intensity projection (MIP) image sets, and their combined volume was used to generate the internal target volume (ITV). There was no expansion for microscopic disease. A 5-mm isotropic margin was added to form the planning target volume (PTV). The radiotherapy plan was calculated on the CT average image set and optimized using 7-10 beam angles. Intensity-modulated radiation therapy (IMRT) was used since 2009. The institutional policy was to deliver 48-52 Gy/4 fractions (fx) for peripheral NSCLC tumors (48 Gy if ≤ 3cm, 52 Gy if > 3cm) and 50 Gy/5fx for all central tumors (defined as tumors immediately adjacent to the esophagus, trachea, main stem bronchi, great vessels, and/or heart), regardless of size or histology. Plans were optimized to aim for ≥ 99% of the ITV to receive the prescription dose (ITV V100 ≥ 99%), and ≥ 99% of the PTV to receive 95% of the prescription dose (PTV V95 ≥ 99%). Radiotherapy plans were corrected for tissue inhomogeneity using the collapsed cone convolution algorithm.

Treatment was delivered using the Elekta Synergy units (Elekta AB, Stockholm, Sweden) equipped with the Elekta Synergy Beam Modulator (high resolution 4 mm multi-leaf collimator), a kilovoltage cone-beam CT (CBCT) image-guidance system and the Hexapod robotic couch permitting 6 degrees of freedom patient positioning.

Radiation was administered in 4-6 fractions (median: 4) with an average biological effective dose of 121 (range: 85.5 - 150).

**Supplementary Table S1. Patient and metastases characteristics.**

| Patients (n) | 111 |
| --- | --- |
| Metastases (n) | 163 |
| Age (mean (range)) | 67 (34 - 90) |
| Sex | 57 F / 54 M |
|  |  |
| Number of metastases per patient |  |
| 1 (n) | 91 |
| 2 (n) | 16 |
| 3 (n) | 2 |
| 4 (n) | 2 |
|  |  |
| Primary Tumor Location |  |
| Colorectal (n) | 88 |
| Lung (n) | 25 |
| Renal cell (n) | 18 |
| Breast (n) | 16 |
| Uterine (n) | 6 |
| Melanoma (n) | 3 |
| Bladder (n) | 3 |
| Head & Neck (n) | 1 |
| Esophageal (n) | 1 |
| Unknown primary (n) | 1 |
| Follow-up period – Median (IQR) days | 927 (677 1232) |
|  |  |
| Clinical outcomes |  |
| Local recurrence | 16 / 111 pts  26 / 163 metastases |
| Recurrence free survival | 28 / 111 |
| Distant recurrence | 72 / 111 |
| Death | 37 / 111 |

**Functional Principal Component Analysis: detailed method**

The Functional Principal Component Analysis (FPCA) is an extension of the classic Principal Component Analysis from the multivariable setting (PCA) to functions, or ‘curves’ (in the current study: the smoothed CT density histograms). While the classic PCA can be used for dimension reduction, the role of the FPCA in the current study is to represent several independent modes of variations of the CT density curves in a patient cohort of interest. On one hand, the classic radiomics features such as skewness or entropy have their origin in the general statistics, information theory or texture analysis literature. On the other hand, the F1, F2, F3 features are data driven, extracted with the FPCA from a sufficiently large sample of lesions from patients having different survival outcomes. The FPCA method first allows us to explore the prominent modes of variation best explaining the variance inside the sample of CT density curves and to use the FPC scores of each lesion to quantify how a specific lesion is situated in the whole lesion population. The scores F1, F2, F3 associated to each CT density curve (one per lesion) are new radiomic variables combined to the other candidate predictors in subsequent multivariate Cox models.

Compared to the PCA operating on p variables using the eigendecomposition of a discrete covariance matrix, FPCA operates on an ‘infinite’ number of dimensions (the points of the CT density curves) and thus uses a smooth covariance matrix. Like in PCA, several eigenvalues $\lambda_{k}$ of descending values are extracted from the covariance matrix but in FPCA, eigenvectors are replaced with eigenfunctions.

In the current study, the FPCs (functional principal components) are extracted through numerical integration since with have a dense series of points along the CT density axis (no sparsity issue).

Thanks to the pivotal Karhunen–Loève theorem, FPCA can transform any (centered) smooth function X(t) (our smooth CT density curve for each metastasis) in an eigenbasis (in FPCA, this is an orthonormal basis made of eigenfunctions in the space of smooth curves)

$$X\left( t \right)-\mu\left( t \right)=\sum_{k=1}^{\infty} \xi_{k}\varphi_{k}\left( t \right) (1)$$

Where $\mu\left( t \right)$ is the mean of our CT density curves, $\varphi_{k}$are the eigenfunctions coming from our eigendecomposition of the covariance matrix and $\xi_{k}$ are the k-th functional principal components (FPCs) computed using a numerical integration. Notice that Variance($\xi_{k}$) = $\lambda_{k}$ (the eigenvalues) and that practically, the infinite sum of equation (1) is replaced with the sum on few dominant functional principal components giving a good approximation of the centered curves: $X\left( t \right)-\mu\left( t \right)$. The functional principal components (FPCs): $\xi_{k}$ are computed such as:

$$\xi_{k}=\int_{\tau} \left( X\left( t \right)- \mu(t))\varphi_{k}(t)dt \right) (2)$$

In practice, when curves are CT density curves, FPCA must be adapted to the specificity of the probability density distributions, with the constraint that their values are always positive and must integrate to 1. Consequently, an appropriate transformation of the curves is applied first (a log quantile density transform) using the function ‘*FPCAdens’* of the ‘fdadensity’ R-library^2^. The same function performs the FPCA described here using the ‘*FPCA*’ function of the of the ‘fdapace’ R-library^3^. Following the authors of the ‘fdapace’ R-library, we called ‘scores’ the estimates of $\xi_{k}$ produced in the ‘FPCA’ function. These scores are used as new variables F1, F2 and F3 in our survival analysis models.

For exploratory purposes, we are mostly interested in representing the modes of variation for each of the k principal components retained (3 FPCs in our study).

$$m_{k,\alpha}\left( t \right)= \mu\left( t \right)\pm\alpha\sqrt{\lambda_{k}}\varphi_{k}\left( t \right)$$

With $\in\tau$ . In our study, the domain $\tau$ is defined from -1000HU to 500HU in the CT attenuation range. $\alpha\in[-A,A]$. In our implementation, to represent the modes of variation for each FPC ‘F1’, ‘F2’ and ‘F3’ (Figure 1 and supplementary figure 1), we plot the mean curve and add multiples to the eigenfunctions based on the following quantiles: 10^th^, 25^th^, 75^th^ and 90^th^. (function ‘*CreateModeOfVarPlotLQ2D*’ of the ‘fdadensity’ R-library).

**Determination of retained number of FPCs**

The determination of the number of FPCs makes use of the Fraction of the Variance Explained (FVE) by the first FPCs using the ‘*GetFVE*’ function of the ‘fdadensity’ R-library^2^ and using the usual Euclidean metric for computing the variances among curves. Several of our published studies with FPCA applied to CT density curves for segmented tumors or other structures showed that only of the very first components have a useful FVE and k=3 is a very acceptable maximum number of components. The usual smoothness of our CT density distribution makes potential higher order principal components improbable significant predictors. In our study, the third FPC ‘F3’ shows a fraction of variance explained already inferior to 10% (7.2%) and F3 was not retained in the final models. Of course, the number of FPC components is highly dependent of the type of curves in the study. For example, it is notorious that FPCA applied to spectra in chemometrics must consider high frequency information and may require a much larger number of FPCs.

**References**

1. Thibault I, Poon I, Yeung L, Erler D, Kim A, Keller B, et al. Predictive factors for local control in primary and metastatic lung tumours after four to five fraction stereotactic ablative body radiotherapy: a single institution's comprehensive experience. Clin Oncol (R Coll Radiol). 2014; 26: 713-719.
2. Petersen A., Müller H-G. Functional data analysis for density functions by transformation to a Hilbert space. The Annals of Statistics. 2016; 44: 183-218.
3. Chen K, Zhang X, Petersen A et al. Quantifying Infinite-Dimensional Data: Functional Data Analysis in Action. Stat Biosci 2017; 9: 582-604.

**
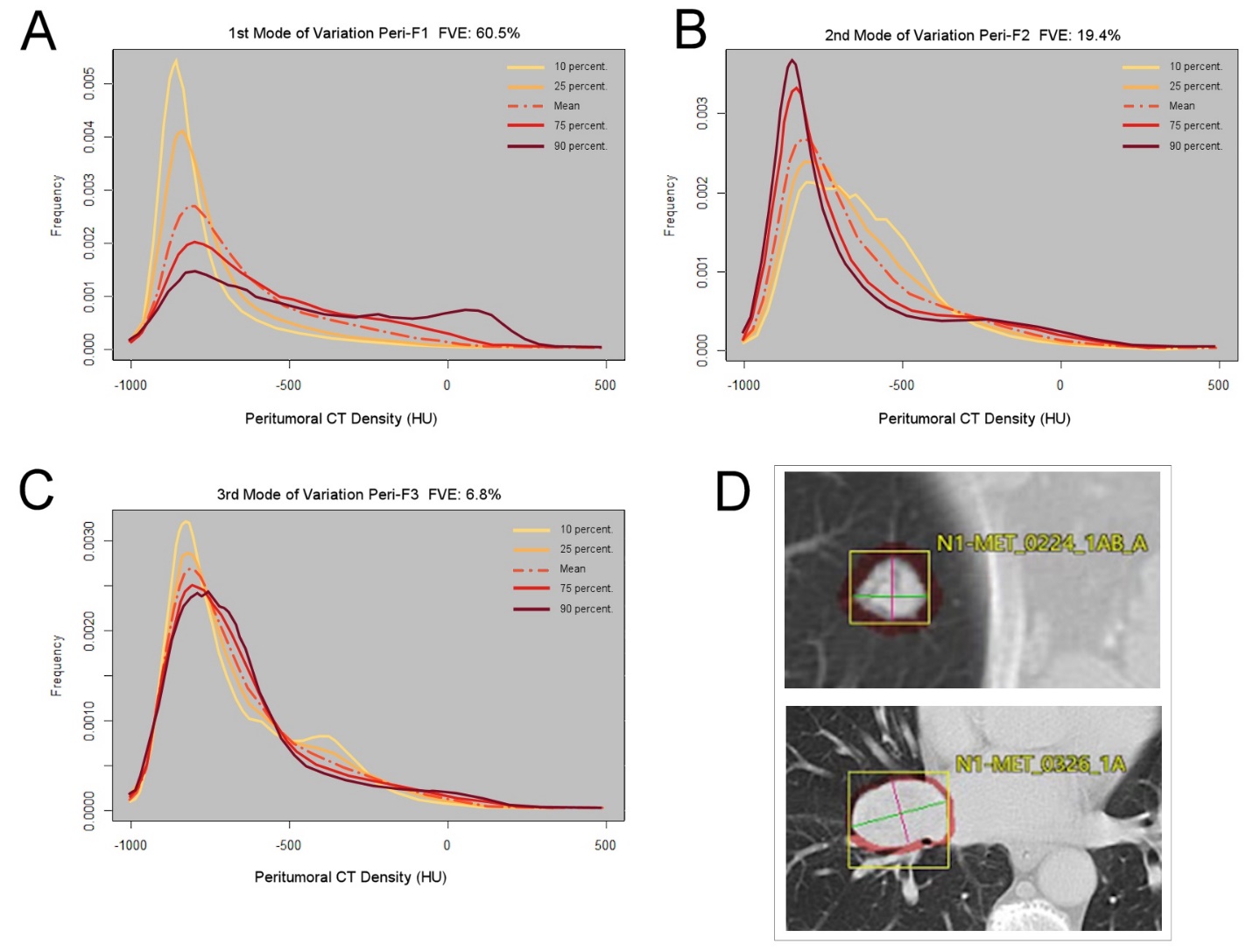
**

**Supplementary Figure S1. Main modes of variation of the peri-tumoral CT density histograms**. A. First main mode variation Peri-F1 of the peri-tumoral CT density histogram from low density homogeneous distribution (yellow) to heterogeneous bimodal distribution (brown). B. Second mode of variation Peri-F2. C. Third mode of variation Peri-F3. D. Example of peri-tumoral region with low Peri-F1 (top) and high Peri-F1 (bottom) (Vitrea software v.7.6, Canon Medical systems, Otawara, Japan).


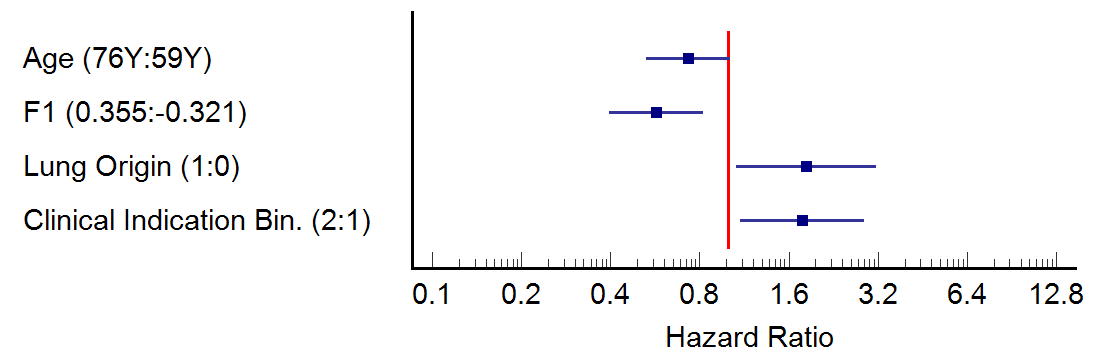


**Supplementary Figure S2. Nomogram for model RFS-3 (with clinical variables).** Hazard ratios are presented with their 95% confidence intervals.


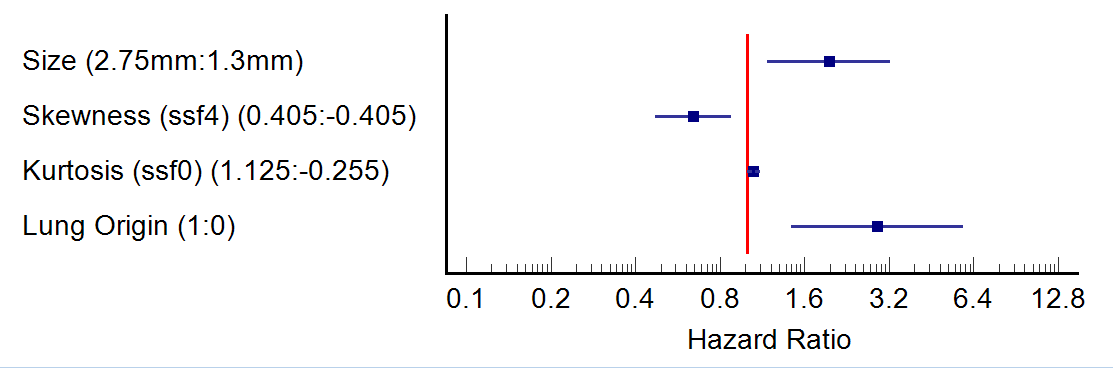


**Supplementary Figure S3. Nomogram for model OS-1 (with clinical variables).** Hazard ratios are presented with their 95% confidence intervals.


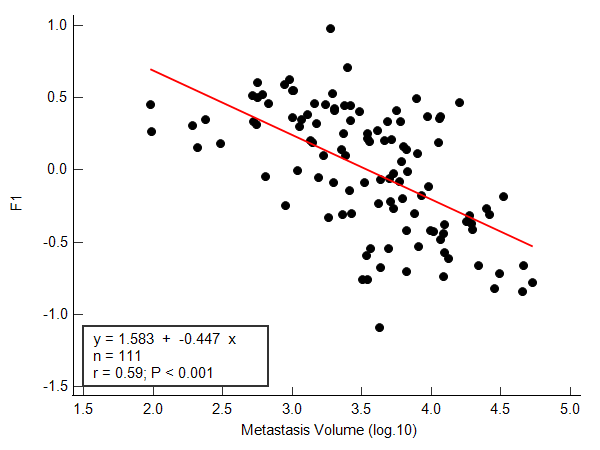


**Supplementary Figure S4. Scatterplot CT density F1 vs. metastasis volume (log) with linear regression line.** The CT density variable F1 appears fairly correlated with the metastasis volume (log). Linear correlation r=0.59.


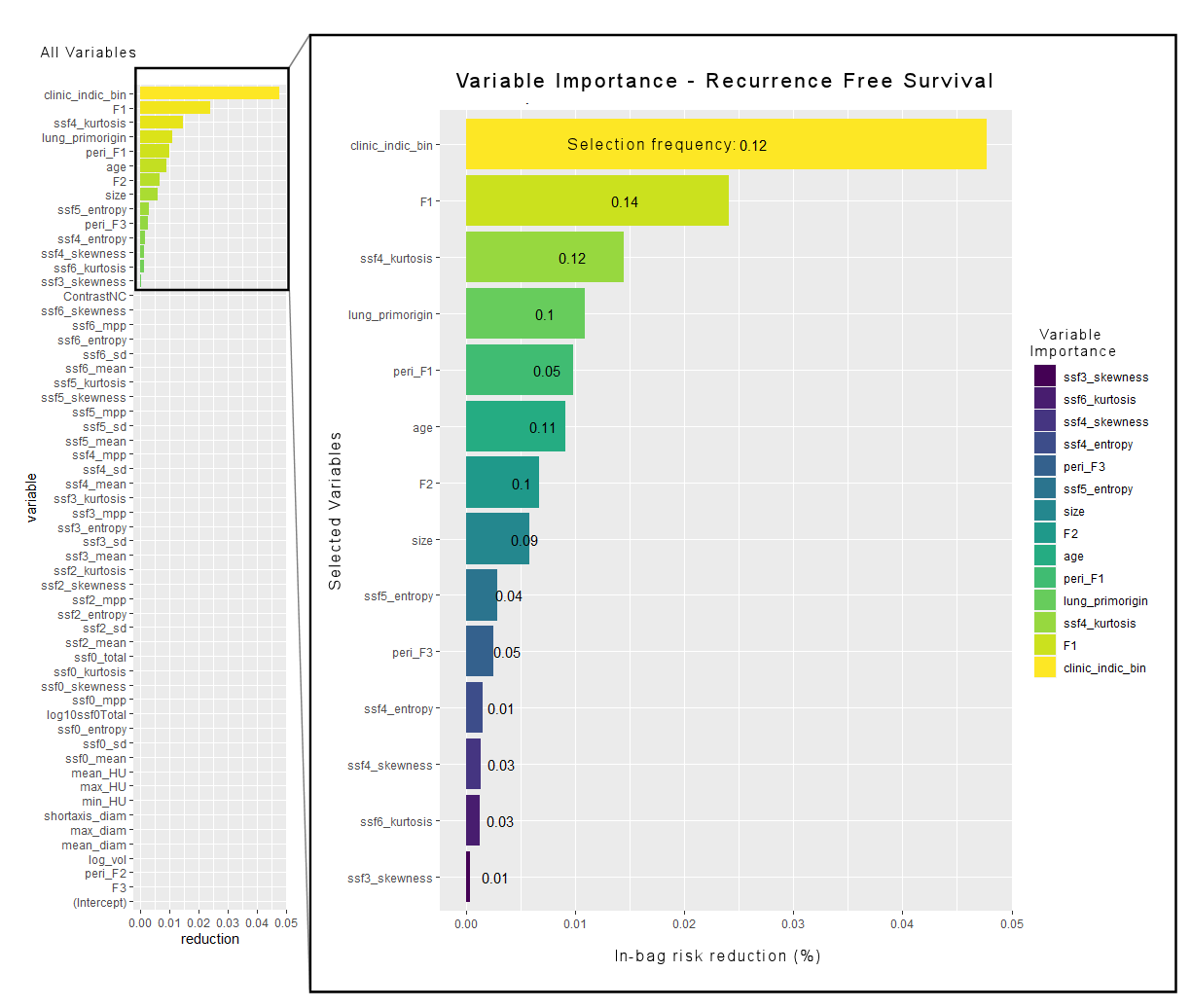


**Supplementary Figure S5. Variable importance for multivariate RFS model variable selection.** Left: original variable list (including clinical variables). Right: Selected variables for Recurrence Free Survival (RFS) multivariate Cox model building after 100 boosting iterations ranked by decreasing importance (% in-bag reduction risk).


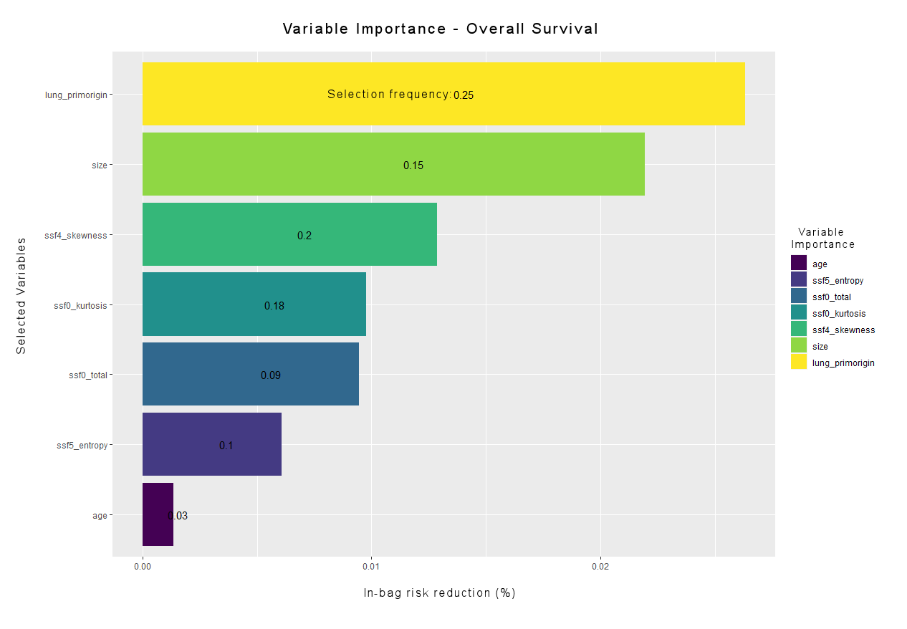


**Supplementary Figure S6. Variable importance (% in-bag reduction risk – 100 iterations) for Overall Survival (OS) multivariate Cox model.**
