# Supplementary material for: Predefined and data-driven CT radiomics predict recurrence-free and overall survival in patients with pulmonary metastases treated with stereotactic body radiotherapy
Source: PLoS One. 2024 Dec 31;19(12):e0311910. doi: 10.1371/journal.pone.0311910 (PMC11687728; doi:10.1371/journal.pone.0311910)
Supplement: Attachment — Submitted filename: Manuscript_Metastases_ResponseToReviewers.docx [file pone.0311910.s008.docx]

Responses to reviewers

**Manuscript: Predefined and data-driven CT radiomics predict recurrence-free and overall survival in patients with pulmonary metastases treated with stereotactic body radiotherapy**

We would like to thank the reviewers for their meaningful comments which have been taken into account for the manuscript revision:

*i) Regarding the C-index values obtained in this study, which range from 0.6 to 0.7, I think the authors should thoroughly discuss this point. It would be beneficial to explore potential strategies for enhancing these values.*

**Response:**

We fully agree with the reviewer’ comments on the C-index performances. The issue on the limited predictive values of our final models is now briefly discussed and acknowledged at the end of the section “limitations” lines 568-572.

*ii) The authors mentioned "univariate (Kaplan-Meier)" in the abstract and main text. However, Kaplan-Meier is not a comparable method. This might refer to "log rank" or a similar statistical test.*

**Response:**

We agree with the reviewer’ comments on the status of the Kaplan-Meier analysis. We removed the “univariate” adjective which seems confusing and added the reference of the log-rank tests which are indeed the inference part of the Kaplan Meier analysis (strictly speaking the Kaplan-Meier curves correspond to the non-parametric survival curve estimator) both in the abstract (‘Results’ section) and in the Discussion – ‘Clinical variables’ section – line 406)

*iii) The administration of adjuvant chemotherapy after SBRT significantly affects RFS or OS. Information on adjuvant chemotherapy should be included for a comprehensive understanding of the treatment outcomes.*

**Response:**

We agree with the reviewer’ comments on the need for information about potential adjuvant chemotherapy.

In our institution, any lung SBRT should NOT be delivered concurrently with adjuvant chemotherapy. A specific mention now appears in the Material and Method section - Follow-up and evaluation of patient clinical outcomes – lines 117-118

________________________________________

Besides these changes: For more study reproducibility, commented R-code scripts with input and output test files corresponding the computation of the Functional Principal Components of the CT histograms for metastases, peri-metastases regions, CT histogram smoothing and others scripts are now available at: <https://github.com/psalaz83/Metastases_Radiomics.git> See also in the same link the README.md file for more explanations on these files.
