# Supplementary material for: Predefined and data-driven CT radiomics predict recurrence-free and overall survival in patients with pulmonary metastases treated with stereotactic body radiotherapy
Source: PLoS One. 2024 Dec 31;19(12):e0311910. doi: 10.1371/journal.pone.0311910 (PMC11687728; doi:10.1371/journal.pone.0311910)
Supplement: Attachment — Submitted filename: Response to Reviewers_Rev2.docx [file pone.0311910.s009.docx]

Response to Reviewers (revision 2)

**Title: Predefined and data-driven CT radiomics predict recurrence-free and overall survival in patients with pulmonary metastases treated with stereotactic body radiotherapy**

We would like to thank the reviewers for their valuable comments. We have tried to the best of our ability to address these comments below. Please note that the Reviewer’s comments have been added in “italic” and any copied and pasted text from the revised manuscript in “Times New Roman”:

i) *Regarding the C-index values obtained in this study, which range from 0.6 to 0.7, I think the authors should thoroughly discuss this point. It would be beneficial to explore potential strategies for enhancing these values.*

*The authors should provide a clearer and more exhaustive response to Reviewer 1's first comment (i).*

*Please elaborate on which could be the actual reasons for C-values between 0.6 and 0.7 and which strategies could be enacted to improve them. In doing so, you should avoid vague terms such as 'notorious difficulty', 'detailed characteristics' (which ones? e.g. tumor biological features? enrollment criteria? patient history? treatment data? other?) and discuss, eventually with reference to the relevant literature and/or current practice.*

Thank you for your insightful comment. We have now included a detailed presentation of our predictive performances (expressed as C-index values) with regards to the most recent literature on predictive models for metastases recurrence and patient survival. Moreover, we elaborated more on the potential most promising directions to improve the existing model performances.

We have expanded the last paragraph of the Limitations section of the Discussion as follows (please see Revised Manuscript - without track changes: lines 588-568):

“Finally, the predictive performances of our best models (C-index: 0.66 and 0.67) for RFS and overall survival are in the range of recent similar studies. For the RFS predictions of patients with colorectal peritoneal metastases undergoing cytoreductive surgery and using clinical variables only, Dietz et al. [57] reported a C-index of 0.64. Sanli et al. [58] reported a predictive survival model with spinal bone metastases with a C-index of 0.669 (0.598–0.740) using composite clinical and radiomic score predictors. Recently, Liao et al. [59] presented a deep learning MRI radiomic model with a C-index of 0.75 to predict survival in patients with brain metastases treated with gamma knife surgery. Based on contrast-enhanced CT for brain metastases, Zhang et al [60] reported a C-index of 0.66 when using both clinical and radiomic predictors. Finally, using contrast-enhanced MRI images, Zhou et al [61] reported a C-index of 0.78 for clinical and volume-based models in patients with brain metastases who were undergoing stereotactic radiosurgery. These performances remain low for definite clinical applicability and should be considered as first steps toward future predictive survival models. Methodologic improvement for better recurrence and predictive survival models are suggested by the recent literature including large dataset prospective studies, homogeneous cancer origins [62], and deep-learning survival models following the predictor selection [59]. Moreover, a one-point radiomic and morphologic analysis ignores most of the dynamic of the cancer progression. Short-term radiomic and morphologic changes may be possible with accurate automatic segmentation, potentially adding predictive power without requiring long follow-up periods. This post-treatment serial follow-up approach may contribute to early recurrence prediction and may be combined with new genetic tests such as serial circulating tumour DNA (ctDNA), recently evaluated for predicting recurrence in colorectal cancer liver metastasis surveillance studies [63].”

ii) *I also suggest improving the English language and style of the manuscript, ideally with the aid of a native English-speaking medical writer or a professional editing service.*

Thank you for your valuable comment and recommendation. We have done our best to edit and improve the manuscript with the help of a professional service and we believe that the revised manuscript has been substantially improved.
